# Supplementary material for: Hepcidin is regulated by promoter-associated histone acetylation and HDAC3
Source: Nat Commun. 2017 Sep 1;8:403. doi: 10.1038/s41467-017-00500-z (PMC5581335; doi:10.1038/s41467-017-00500-z)
Supplement: Supplementary file 1 — Supplementary Information [file 41467_2017_500_MOESM1_ESM.pdf]

Description of Supplementary Files

File name: Supplementary Information
Description: Supplementary figures and supplementary tables.

File name: Peer review file

Supplementary Figure 1

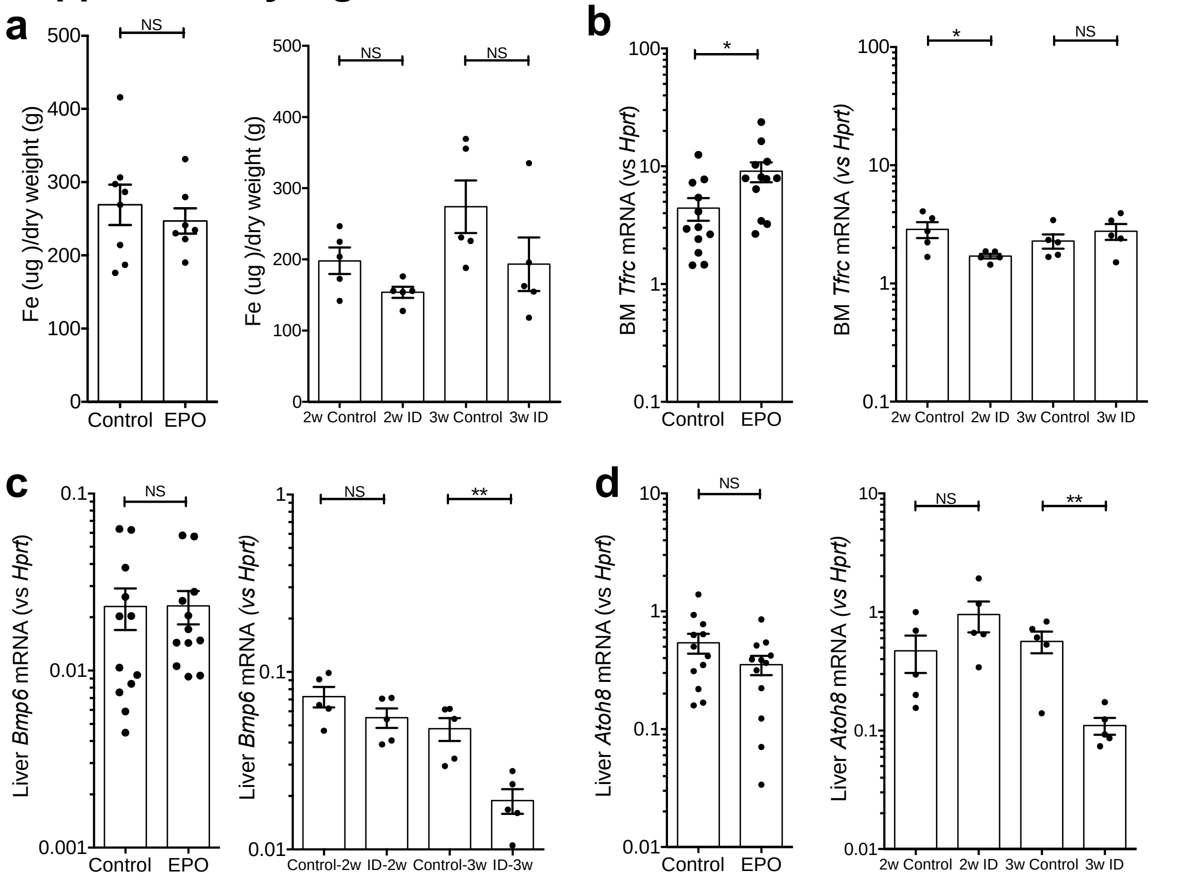

Supplementary Figure 1. Effects of 3d Epo 200iu compared with control in 6w C57BL/6 male mice, and 2 week low iron diet in 4w C57Bl/6 mice, on (a) liver non-heme iron content (N=8 for liver iron for Epo experiments, N=5 for liver iron for ID experiments) , (b) bone marrow Tfrc mRNA, (c) liver *Bmp6* mRNA, and (d) liver *Atoh8* mRNA. Epo experiments; iron deficiency experiments. Student t-tests. Data are means +/- s.e.m. *p≤0.05; **P≤0.01; ***P≤0.001; ****P≤0.0001; NS – P>0.05.

Supplementary Figure 2

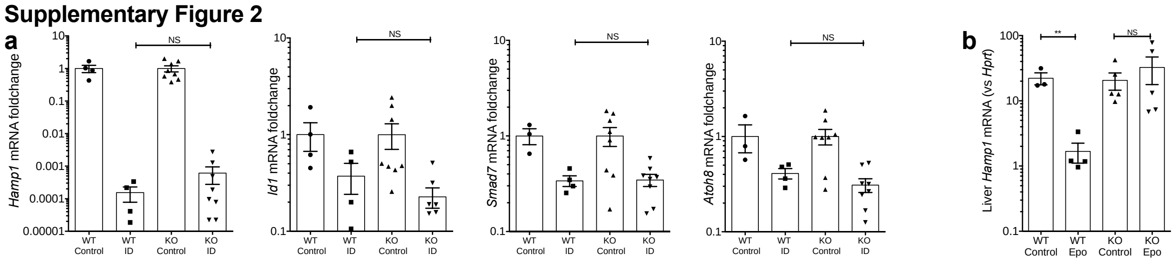

Supplementary Figure 2. (a) Effects of 3 week iron deficient diet on foldchange *Hamp1*, *Id1*, *Smad7* and *Atoh8* mRNA expression in 5 week old WT and *Fam132b* knockout mice. (b) Effects of single dose Epo 200iu i.p. in wildtype and *Fam132b* knockout mice on *Hamp1* mRNA expression. Student t-tests. Data are means +/- s.e.m. *p≤0.05; **P≤0.01; ***P≤0.001; ****P≤0.0001; NS – P>0.05.

Supplementary Figure 3

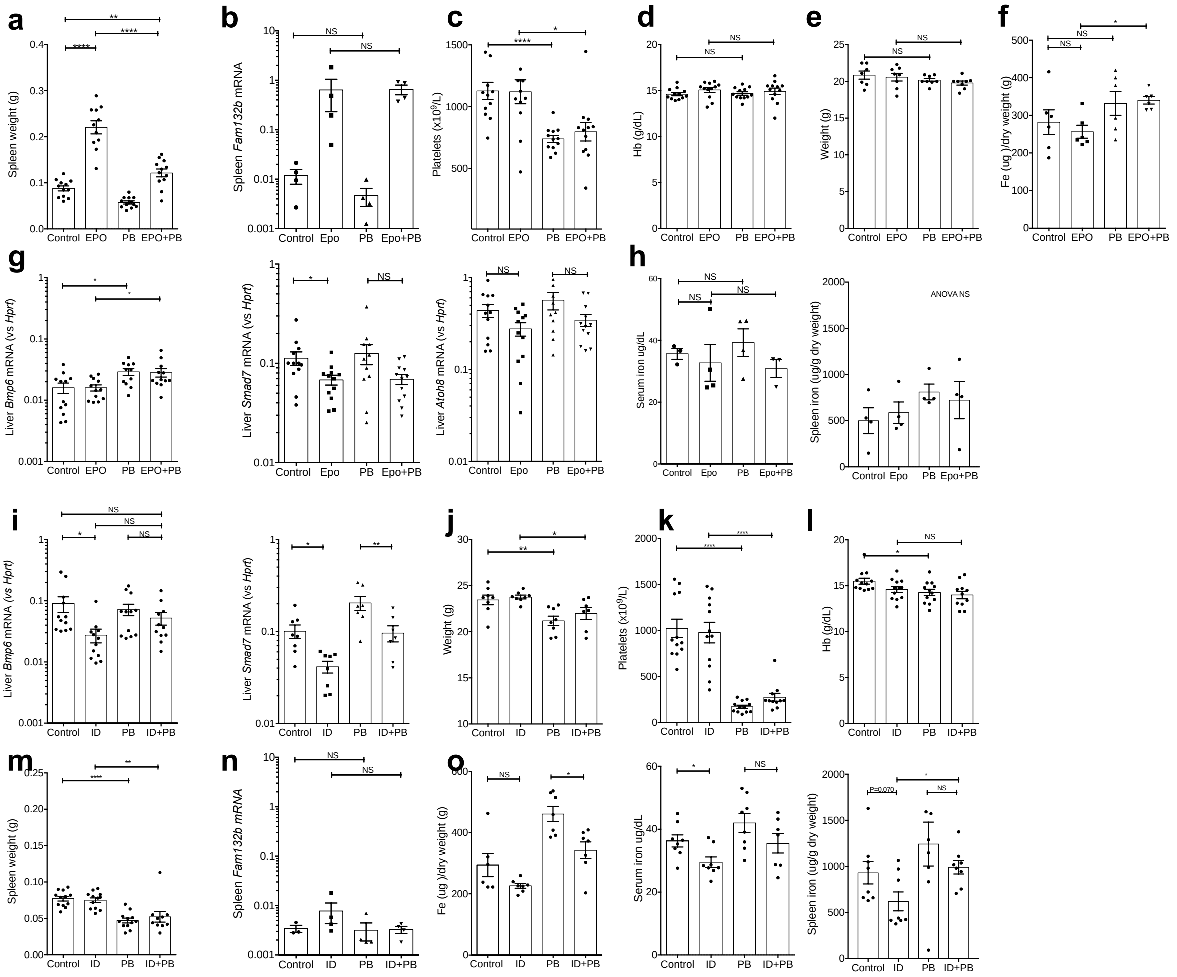

Supplementary Figure 3. Effects of 200IU Epo per day x 3d with or without Panobinostat 0.4mg (20mg/kg) x 3d in 6w old C57Bl/6 male mice. Three identical experiments each comprising 4 mice per group were performed, and are presented here as combined data. (a) Spleen weight, (b) spleen *Fam132b* mRNA expression, (c) platelet count, (d) hemoglobin concentration, (e) mouse body weight, (f) non-heme liver iron content, (g) liver *Bmp6*, *Smad7* and *Atoh8* mRNA expression, and (h) serum and spleen iron (N=4 per group). Effects of 2 weeks low iron diet with or without Panobinostat 0.4mg (20mg/kg) x 7d in 6w old C57Bl/6 male mice. Three identical experiments each comprising 4 mice per group were performed, and are presented here as combined data. (i) Hepatic *Bmp6* and *Smad7* mRNA expression, (j) mouse body weight, (k) platelet count, (l) hemoglobin concentration, (m) spleen weight, (n) spleen *Fam132b* mRNA expression, and (o) non-heme liver iron content and serum and spleen iron (n=8 per group). Data are means +/- s.e.m. Student t-tests. Data are means +/- s.e.m. *p≤0.05; **P≤0.01; ***P≤0.001; ****P≤0.0001; NS – P>0.05.

Supplementary Figure 4

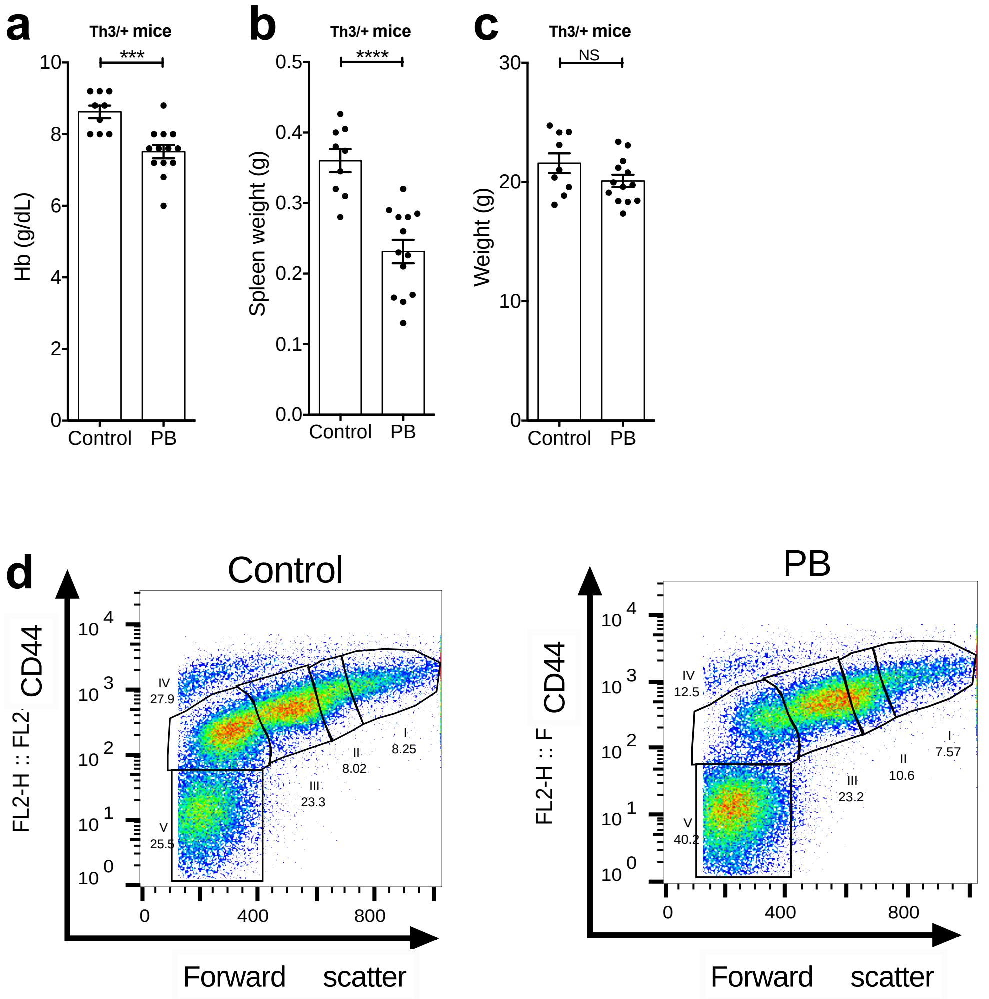

Supplementary Figure 4. Effects of 5mg/kg/d Panobinostat (PB) in Th3/+ mice, on (a) Hemoglobin concentration, (b) spleen weight, (c) body weight, and (d) erythroblast maturation – flow cytometry of splenocytes (CD44 vs Forward Scatter). Data are means +/- s.e.m. *p≤0.05; **P≤0.01; ***P≤0.001; ****P≤0.0001; NS – P>0.05.

Supplementary Figure 5

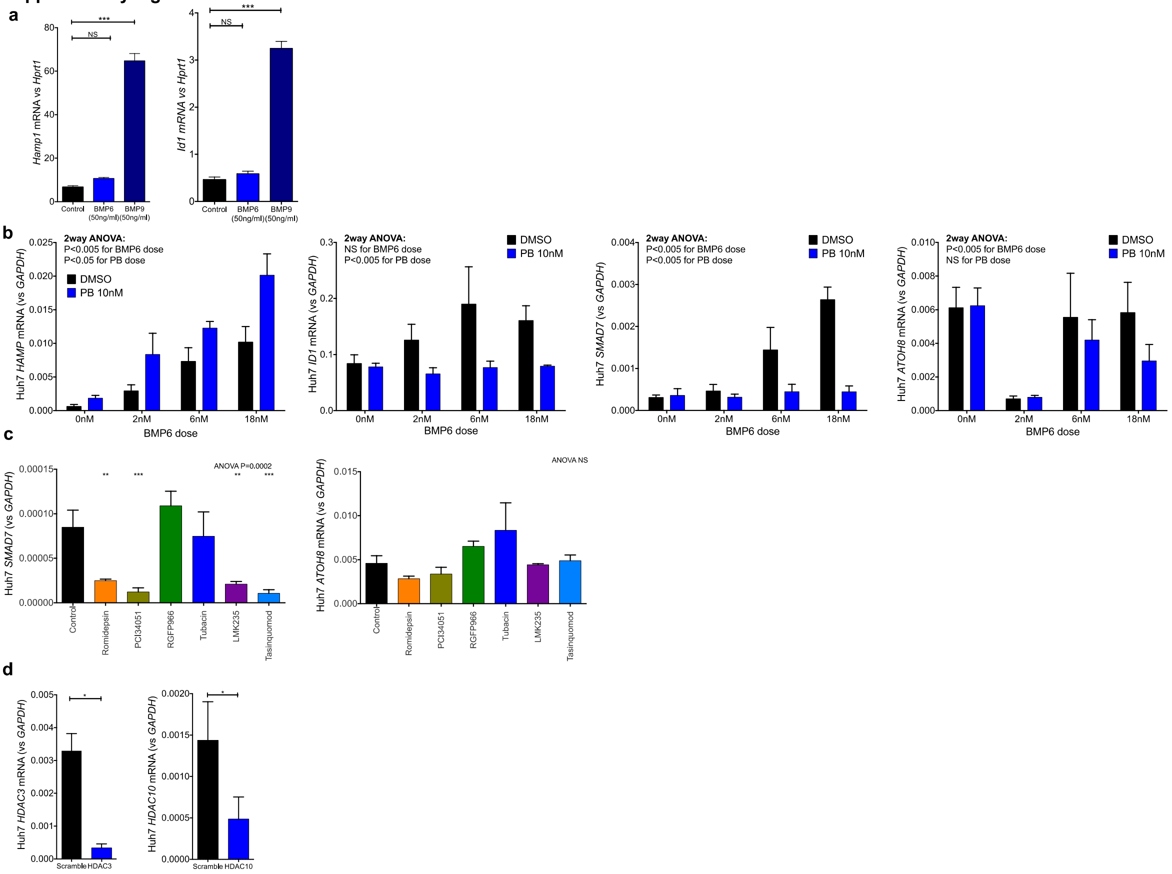

Supplementary Figure 5. (a) Effect of treatment of precision-cut liver slices with recombinant human BMP6 and BMP9 (both 50ng/mL), on *Hamp1* and *Id1* mRNA expression. (b) Effects of treatment of Huh7 cells with BMP6 and PB on *HAMP1*, *ID1*, *SMAD7*, and *ATOH8* mRNA expression (n=3, two-way ANOVA). (c) Effects of treatment of HUH7 cells with HDAC specific inhibitors on *SMAD7* and *ATOH8* mRNA expression (n=3, one-way ANOVA and paired t-test for differences from control). (d) Validation of results of siRNA screen: knockdown of HDAC3 and HDAC10; n=3 per group, paired t-test. Data are means +/- s.e.m. *p≤0.05; **P≤0.01; ***P≤0.001; ****P≤0.0001; NS – P>0.05.

Supplementary Figure 6

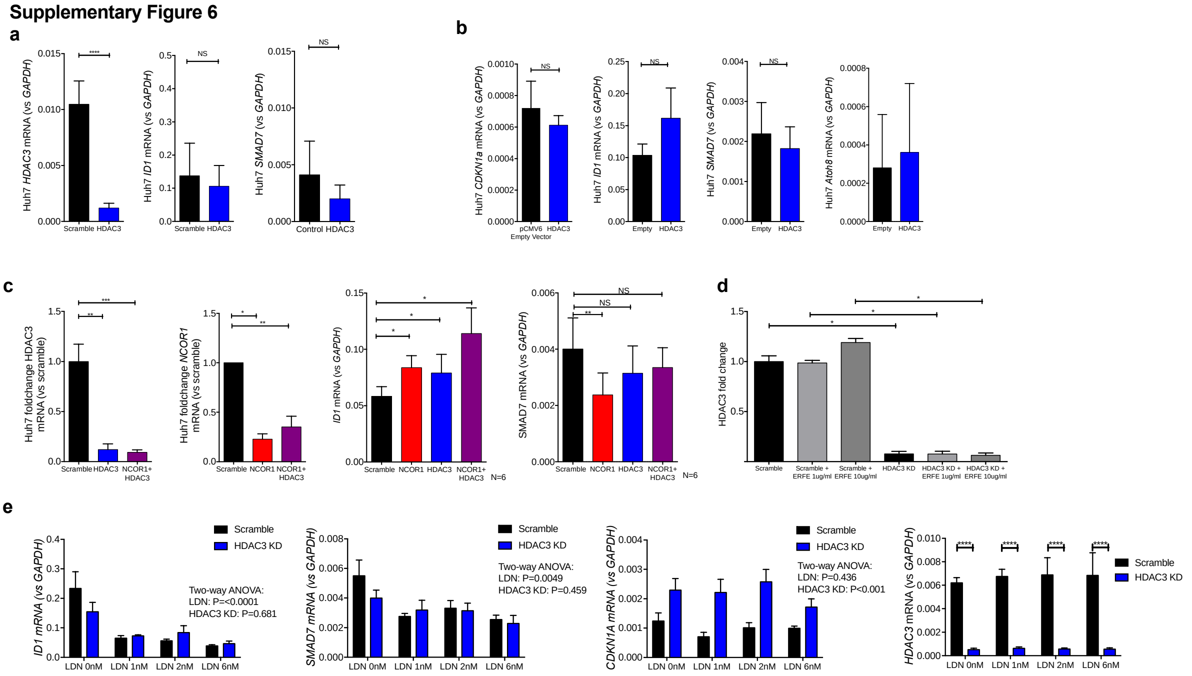

Supplementary Figure 6. (a) Effects of scramble or HDAC3-specific siRNA in Huh7 cells on *HDAC3* mRNA expression; *ID1* mRNA, and *SMAD7* mRNA expression; paired ratio t-tests. (b) Effects of HDAC3 overexpression in Huh7 cells on *CDKN1A*, *ID1*, *SMAD7* and *ATOH8* mRNA expression; paired t-tests. (c) Effects of siRNA against HDAC3 or NCOR1 individually or in combination on *HDAC3* mRNA expression and *NCOR1* mRNA expression, as well as *ID1* and *SMAD7* mRNA expression in Huh7 cells; paired ratio t-tests. (d) Effects of siRNA against HDAC3 in Erfe co-treatment experiments on *HDAC3* mRNA expression. (e) Effects of HDAC3 siRNA +/- LDN13189 on Huh7 *HDAC3*, *ID1*, *SMAD7* and *ATOH8* mRNA expression (two-way ANOVA). Data are means +/- s.e.m. *p≤0.05; **P≤0.01; ***P≤0.001; ****P≤0.0001; NS – P>0.05.

Supplementary Figure 7

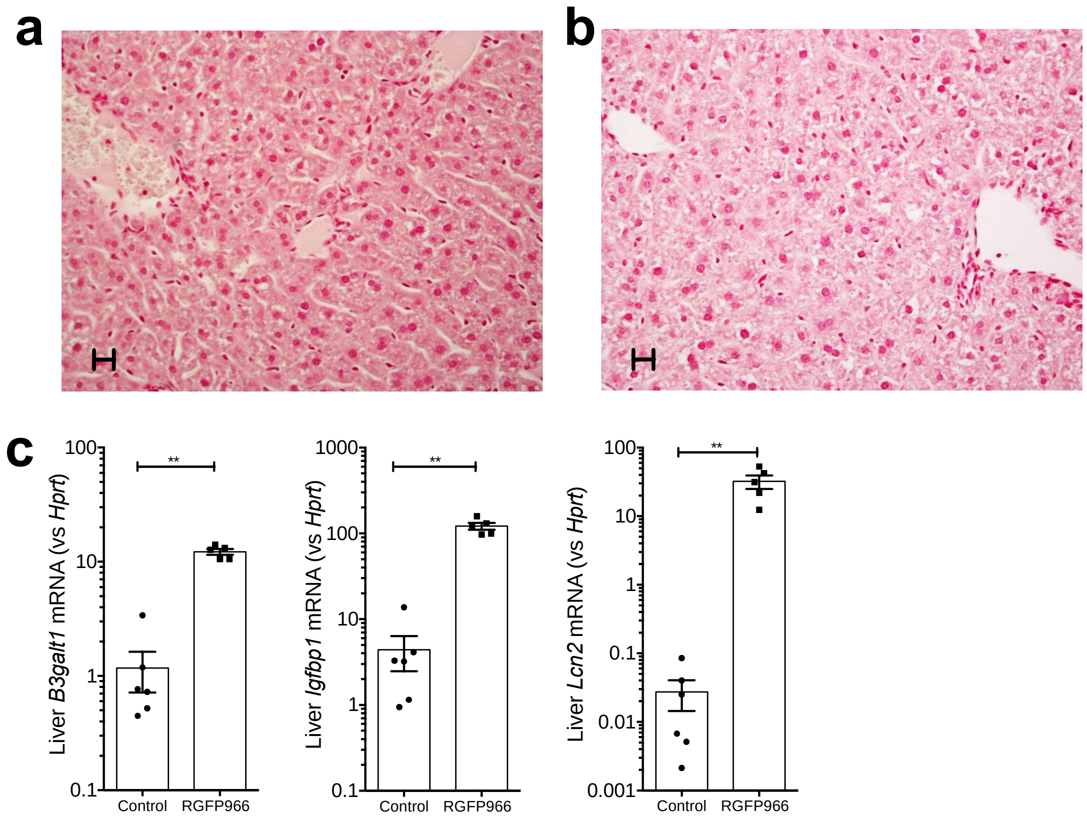

Supplementary Figure 7. (a) C57Bl/6 mice received a 3 week low iron diet, followed by two doses of RGFP966 20mg/kg/dose. Perl's stain of livers from control mice; (b) Perl's stain from mice receiving RGFP966. (N=2 mice per group); size marker 10um. (c) Gene expression from control and Epo treated mice used for HDAC3 ChIP-qPCR (t-test). (d) Validation of top differentially expressed genes identified by RNA-Sequencing from *in vivo* RGFP966 experiment described in (a) and (b) above – *B3galt1*, *Igfbp1*, *Lcn2* (N=6 per group, t-test) **P≤0.01.

Supplementary Figure 8

Figure 6e

H3K9ac

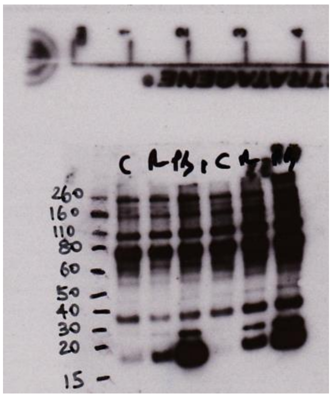

Actin

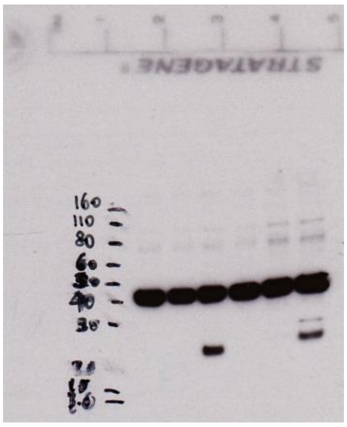

Figure 8h

Ferroportin

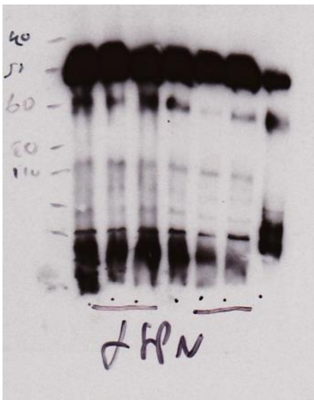

Actin

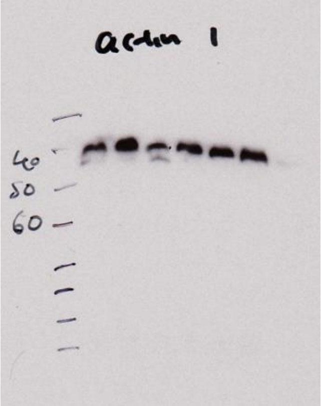

Supplementary Figure 8. Uncropped Western blots of images shown in Figures 6e and 8h.

Supplementary Table 1: Assays used for measurement of gene expression by qRT-PCR

**Taqman gene expression**	
**Gene**	**Taqman Gene Expression Product ID**
**Mouse**	
Hprt1	Mm01545399_m1
Hamp1	Mm04231240_s1
Id1	Mm00775963_g1
Bmp6	Mm01332882_m1
Smad7	Mm00484742_m1
Atoh8	Mm00464055_m1
Fam132b	Mm00557748_m1
Tfrc	Mm00441941_m1
Glyc	Mm01158732_m1
Cyp2b20	Mm00456591_m1
Hdac1	Mm02745760_g1
Hdac2	Mm00515108_m1
Hdac3	Mm00515916_m1
Hdac4	Mm01299557_m1
Hdac5	Mm01246076_m1
Hdac6	Mm00515945_m1
Hdac7	Mm00469527_m1
Hdac8	Mm01224980_m1
Hdac9	Mm01293999_m1
Hdac10	Mm01308119_g1
Hdac11	Mm01183513_m1
B3galt1	Mm00480352_s1
Igfbp1	Mm00515154_m1
Lcn2	Mm01324470_m1
**Human**	
GAPDH	Hs99999905_m1
HAMP	Hs00221783_m1
ID1	Hs00357821_g1
SMAD7	Hs00998193_m1
ATOH8	Hs01031629_m1
HDAC3	Hs00187320_m1
NCOR1	Hs01094541_m1
CDKN1A	Hs00355782_m1
**SYBR Green Gene Expression**	
**Gene**	**Forward**<br>**Reverse**
GAPDH	catgagaagtatgacaacagcct<br>agtccttccacgataccaaagt
HAMP	ctctgttttcccacaacagac<br>taggggaagtgggtgtctc
HDAC3	gacctatgacaggactgatgagg<br>gaactcattgggtgcctctg
HDAC10	tgggaagctcctgtacctctt<br>ggctggagtggctgctatac

Supplementary Table 2: Primers used for measurement of enrichment of genomic DNA by ChIP-qPCR

**Region**	**Forward 5' To 3'**	**Reverse 5' To 3'**	
**Mouse**			
-600bp *Hamp1* Promoter	gcattggctctgcctatgat	cgtggagaccactgtgaaga	
-500bp *Hamp1* Promoter	cagggctaatgctgacacaa	tctcagaaggacccagcaat	
-400bp *Hamp1* Promoter	acattgctgggtccttctga	tggattggtgagtggaagaac	
-300bp *Hamp1* Promoter	aggggaaagaaggggaattt	acagaaccagggacacaagc	
-200bp *Hamp1* Promoter	tgtcactgttcccgcttatc	acccccattttgctctgac	
Exon1 *Hamp1*	accacctatctccatcaacagg	ccatcactcctgagccattc	
Intron1 *Hamp1*	cgtcagggcattctttaagc	agaatactttgcctggctgtc	
Exon2 *Hamp1*	ccgggtactccacaagattc	tgctcttaccgcaatgtctg	
Exon3 *Hamp1*	gctaggctgcttacctctctttc	agatgcagatggggaagttg	
Hprt1 Promoter	tctagaaggagcttcggttcc	tggcatctgcagaattaggg	Positive control H3K9ac, H3K4me3, RNA pol ii, Negative control H3K27me3
Gene Desert on Chr7	ataaaggcttggcactcgtc	cagttccctttgcttgatcc	Negative control H3K9ac, H3K4me3, RNA pol ii
Cdkn1a locus	gggtacagcctcgttcctg	atactgtgcccgccaaatag	Positive control for HDAC3
Actb2	Active Motif Mouse Positive Control Primer Set Part 71017		Positive control for RNA pol ii.
Pax2	Active Motif Mouse Positive Control Primer Set Part 71020		Positive control for H3K27me3
Human			
HAMP locus	ctgttttcccacaacaggtg	ctcagtgctcgggtgtctc	
GAPDH	tccaattccccatctcagtc	agcaggacactagggagtcaag	
Negative region	ccaacaacacacttggatcag	tctgggagaattcagctgtg	
